# SFBC Recognition over Orthogonal Frequency Division Multiplexing Schemes in the Presence of Inphase and Quadrature Phase Discrepancies for Cognitive Radio Applications

**DOI:** 10.3390/s23115267

**Published:** 2023-06-01

**Authors:** Mohamed Marey, Ahmed Sedik, Hala Mostafa

**Affiliations:** 1 Smart Systems Engineering Laboratory, College of Engineering, Prince Sultan University, P.O. Box 66833, Riyadh 11586, Saudi Arabia; sedik@psu.edu.sa; 2 Department of the Robotics and Intelligent Machines, Faculty of Artificial Intelligence, Kafrelsheikh University, Kafrelsheikh 33516, Egypt; 3 Department of Information Technology, College of Computer and Information Sciences, Princess Nourah bint Abdulrahman University, P.O. Box 84428, Riyadh 11671, Saudi Arabia

**Keywords:** signal recognition, SFBC, maximum likelihood estimation

## Abstract

A radio is adaptive if it can autonomously analyze the communications environment and instantly modify its settings to achieve the best possible efficiency. In orthogonal frequency division multiplexing (OFDM) transmissions, identifying the space frequency block coding (SFBC) category utilized is one of the most important tasks of an adaptive receiver. Previous approaches to this problem did not take into consideration the fact that real systems typically suffer from transmission defects. This study offers a novel maximum likelihood recognizer capable of distinguishing between SFBC OFDM waveforms in the context of inphase and quadrature phase differences (IQDs). The theoretical findings show that IQDs arising from the transmitter and recipient can be combined with channel paths to generate so-called effective channel paths. The conceptual examination demonstrates that the outlined maximum likelihood strategy of the SFBC recognition and effective channel estimation processes is implemented by an expectation maximization tool utilizing the error control decoders’ soft outputs. The simulations results reveal that the suggested strategy delivers a much greater recognition accuracy than the typical approaches outlined in the comparable literature. At a signal-to-noise ratio (SNR) of 14 dB, for example, the proposed approach achieves a bit error rate (BER) of 0.00002, which is very close to the case of perfect estimation and compensation for IQDs, outperforming the previous reported works which achieved BERs of 0.01 and 0.02.

## 1. Introduction

The increased demand for high speed data transfers, in conjunction with the limited availability of the licensed spectrum, has fueled the explosive expansion of the radio communications market in recent years, and this progress is expected to accelerate over the coming decade. The deployment of adaptive transmissions is indispensable in order to meet this demand [1,2]. Waveform recognition (WR) is the process of analyzing a collected waveform in order to investigate its properties. It is the central operation of adaptive radios, which modify the broadcast parameters in response to channel state information. This optimizes the service level while reducing the power consumption and boosting the information rate during transmissions. The concept of WR is currently being utilized in a vast array of circumstances, including in the armed services, government, and private industry [3].

The tactical utilization of WR was the inspiration for its advancement, as threat evaluation and digital warfare require the recognition of the broadcast parameters of an intercepted waveform to differentiate enemy emitters, produce blocking waves, and retrieve the captured signal [4,5]. Examples of the broadcast characteristics include modulation and coding types, information rate, and carrier frequency. The broadcasters of the majority of current industrial applications, such as cellular and microwave communications systems, select their parameters from a collection of options available to both the transmitting and receiving sites. Due to the fact that the receiver is unaware of the precise parameters that were applied during transmission, the receiving station is required to implement various kinds of WR mechanisms to secure the truthfulness of the demodulated information. Cognitive radios provide another illustration of the importance of WR, as they enable unauthorized individuals to send information over channels that are already being utilized by other lawful customers. A WR method is a critical component of the architecture of cognitive radios, and its principal intent is to evaluate the type of parameters available in the collected waveforms [6,7]. This allows cognitive radios to transmit without interfering with customers who are presently accessing their channels. Government bodies also employ WR for spectrum surveillance to guarantee conformity to radio licensing requirements. This ensures that crucial organizations, such as security services, firehouses, navigation stations, and armed forces, have access to powerful communication systems that are immune to environmental interference [8,9,10].

In general, the majority of modern wireless systems need to transmit massive volumes of data via wideband frequency selective links. In such environments, it is crucial that undesirable inter-symbol disturbance is not incorporated into the system design. Multicarrier transmission systems have been demonstrated to be an appropriate solution to mitigating the detrimental effects of such channels [1,2,11]. The fundamental idea behind such setups is to divide a high speed serialized data flow into many simultaneous low speed sub-flows. Each sub-flow is processed by a single sub-carrier, resulting in a lower susceptibility to inter-symbol disturbance and, as a consequence, a simplified equalization mechanism. Meanwhile, multiple-input multiple-output technology has been developed as a further advance in the development of wireless communication systems. This is because of the significant performance benefits in linking dependability, data throughput, and broadcast distance, as well as the ease with which it is implemented. It comes as no surprise that several of the world’s current communications systems have incorporated the combination of multicarrier and multiple-input multiple-output technologies. Examples involve digital audio and video broadcast systems, wireless local area connections, satellite and microwave systems, as well as the fourth and fifth generations of cellular communications [12].

When compared to the conventional superheterodyne topology, the direct conversion structure is more appealing as the front-end of multicarrier schemes due to its compact size and ease of integration on a single chip [13]. Furthermore, it offers excellent adaptability to meet the evolving needs of today’s communication infrastructures for radio standards. The discrepancy between the in-phase (I) arm and the quadrature-phase (Q) arm, which can occur at both the broadcaster and recipient, is one of the key difficulties with this structure. This IQ discrepancy (IQD) is common in analog computation because of component flaws, which cannot be anticipated or managed and tend to widen as production technologies shrink in size [14,15]. Tolerating these abnormalities in the analog domain and correcting for them digitally is simpler and more versatile than reducing the IQD with costly analog devices. The IQD, specifically, can be broken down into two distinct types: frequency-flat and frequency-selective IQD [16]. The former is mainly produced by poorly balanced local oscillators, which fail to generate I and Q arms with identical amplitudes and a precise 90 degree phase shift. The latter is normally brought about by other defective analog parts including filters, amplifiers, and converters from digital to analog or analog to digital.

Multicarrier schemes are more susceptible to IQDs than single-carrier transmissions are. This is because the sub-carriers in multicarrier schemes have spectral clash and tight separation between them. As a result, sub-carrier orthogonality of multicarrier schemes breaks down, inter-carrier disturbance grows, and the overall efficiency suffers. The impact, assessment, and correction of the IQD for multicarrier communications have been discussed in a significant number of studies, see [17,18] and the citations therein.

### 1.1. Related Works

Many aspects of WR have been the subject of extensive research, including the recognition of modulators [19,20,21,22], channel control encoders [23,24], multi-carrier broadcasts [1,2,12], and space coding emissions [25,26,27,28,29]. The following discussion delivers a background on latest developments in the subject of space coding recognition, which is the principal focus of this investigation.

The Alamouti (AL) and spatial multiplexing (SM) waveforms over Nakagami links were detected by employing a suite of approaches that rely on higher-order statistics [25]. These methods relied on the fourth-order moment as an identifying feature and required a recipient that was only outfitted with a single antenna. By examining the cyclostationarity of the waveforms coming from two antennas, it was possible to distinguish between multiple space time block code (STBC) waveforms even when experiencing a wide range of broadcast problems [26,27]. The authors of [28] distinguished between SM and AL waveforms by analyzing the dispersion properties of multiple tapped radio channels. It was demonstrated that the correlation function of two collected waveforms has spikes at a specific group of lags when AL was used, but not if SM was utilized. Two different recognition methods were proposed, using maximum likelihood and fake alarm probability criteria as their foundations. Three maximum likelihood mechanisms were devised for blind recognition of STBC waveforms under the presumption of flawless synchronization at the receiver terminal [30]. With precise clock alignment, a complete rank-channel, and an equal or higher number of receiver antennas than transmitting antennas, it was shown in [31] that the Frobenius norms of certain statistics have non-zero characteristics whose positions solely rely on the STBC employed at the transmitter end. This was put to use in labeling five different STBC waveforms. A convolutional neural network with a multiple delay characteristics fusing technique was used to intelligently distinguish STBC signals, as described in [32]. The properties of several time lags were incorporated using two different fusion methods, and a residual block was used to give additional discriminatory features. The authors of [10] presented a categorization technique that analyzed the nature of the AL signals in order to automatically determine the modulation format of the incoming signal. The algorithm classified various modulation types in the presence of carrier phases and frequency offsets without requiring any previous knowledge about channel coefficients or time offsets. This was accomplished by making use of the statistical characteristics of a correlation function between two AL signals that have been collected by multiple antennas. The problem of STBC recognition was discussed in [33,34] for orthogonal frequency division multiplexing (OFDM) schemes across multiple taps links. Using the redundancy founded in space and time, a dual hypothesis assessment for making choices was developed. Analysis of the correlation functions of waveforms collected from a couple of antennas allows for discrimination between space frequency block code (SFBC) signals [29]. The process included two stages. The first step included estimating the correlation function of a couple of waveforms collected from different antennas, and the second step involved using a false-alarm-dependent evaluation for efficient decision making. The authors of [35] explored the problem of STBC detection and channel prediction for multiple-user asynchronous uplink broadcasts in single carrier frequency division multiple access networks. The space alternating generalized expectation maximization approach was utilized to implement a recursive strategy for STBC recognition, channel prediction, and synchronization. The authors of [36] presented a sequential strategy to develop a maximum likelihood predictor that could discriminate between SFBC waveforms across unknown radio links using the channel decoders’ outputs. Across unknown multiple taps links, the authors of [37] concurrently detected modulation types and STBC configurations without requiring more antenna components at the receiver than the broadcaster. Theoretical methods demonstrated that a recurrent expectation maximization strategy with the supplementary process of channel awareness yielded a maximum likelihood solution.

### 1.2. Novelty and Contributions

The previous works of WR were carried out without taking into account the transmission limitations of IQDs. Nevertheless, IQDs are always present in real-world wireless deployments. The key remarkable elements and contributions of this work are listed here.

- We will design a novel SFBC recognition algorithm for deployment in OFDM networks when IQDs are present. Developing a channel estimator will be an integral service we will adopt to enable the recognition process.
- We will consider that IQDs will take place at both the transmitter and recipient. The overall channel taps will be determined by incorporating the effects of IQDs at both endpoints into the actual link paths.
- This work’s analytical investigation will illustrate that the exact maximum likelihood strategy in SFBC waveform recognition of OFDM systems will be too expensive for practical deployment. As a consequence, we will apply the expectation maximization algorithm as an innovative and cost-effective iterative technique.
- The proposed recognizer will leverage the a posteriori probabilities of the conveyed symbols provided by a channel control decoder to assess the a posteriori expectations of the broadcast symbols, which will be processed as if they are training data.

The following is a list of the many benefits that the suggested approach offers.

- It is consistent with any error control decoder mechanism that can determine the soft information of the broadcast symbols. This involves convolutional, turbo, and low density parity check decoders.
- It applies to any collection of SFBC signals, irrespective of the nature of their broadcast patterns, which include the number of transmit antennas and subcarriers employed in a space frequency block.
- It keeps up a reliable performance while maintaining the computational overhead within reasonable bounds.

The preceding is a description of the upcoming tasks. Section 2 describes the system structure and the challenge. Section 3 discusses the proposed maximum likelihood recognizer along with its practical deployment. Section 4 outlines the simulation findings as well as pertinent interpretations. Section 5 introduces the concluding remarks of the work along with the possible future tasks.

## 2. System Structure and Problem Formulation

**Figure 1:**
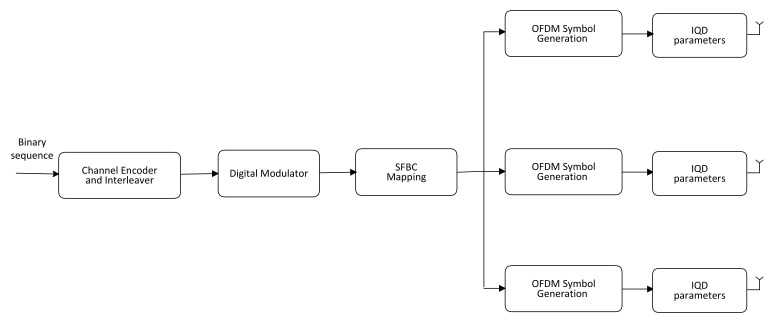
Schematic of an SFBC OFDM transmitter.

We consider SFBC OFDM broadcasts with *N* subcarriers, ν cyclic prefix (CP) samples, *F* broadcast antennas, and a receiver antenna. Figure 1 depicts the notional schematic diagram of a broadcaster. An error control encoder and an interleaver work together to secure a binary stream.

An interleaver spreads out the sequence of bits in a bit stream to offer time diversity by minimizing the effect of transmission errors such as bursts. The encoded information is converted into data symbols by a digitized modulator, with each symbol being selected from an *M*-point signal pattern, Ω. A vector $D=\left[d_{0},d_{1},\cdots ,d_{U-1}\right]$ of length *U* is formed by combining a number of training symbols with data information. There are no limitations placed on the channel coding, interleaver, mapping technique, pilots’ allocation, or constellation type that may be used in this scenario. Separate chunks of length *P* are created from the components of vector D. The encoder of SFBC signals utilizes a predetermined F×Q code matrix $C\left(d^{\left(p\right)}\right)$ to distribute the *p*th chunk, $d^{\left(p\right)}$, over *Q* consecutive subcarriers for transmission through *F* antenna elements. For clarification, the broadcast matrices of SFBC1 $\left(P=2,Q=1,F=2\right)$, SFBC2 $\left(P=2,Q=2,F=2\right)$, SFBC3 $\left(P=3,Q=4,F=3\right)$, and SFBC4 $\left(P=4,Q=8,F=4\right)$ are [38]:

(1) $$C^{\left(\mathrm{SFBC}1\right)}\left(d=\left[d_{0}\;d_{1}\right]\right)={\left[d_{0}\;d_{1}\right]}^{T},$$

(2) $$C^{\left(\mathrm{SFBC}2\right)}\left(d=\left[d_{0}\;d_{1}\right]\right)={\left[\begin{array}{cc} d_{0} & d_{1}\\ -d_{1}^{*} & d_{0}^{*} \end{array}\right]}^{T},$$

(3) $$C^{\left(\mathrm{SFBC}3\right)}\left(d=\left[d_{0}\;d_{1}\;d_{2}\right]\right)={\left[\begin{array}{ccc} d_{0} & d_{1} & d_{2}\\ -d_{1}^{*} & d_{0}^{*} & 0\\ d_{2}^{*} & 0 & -d_{0}^{*}\\ 0 & -d_{2}^{*} & d_{1}^{*} \end{array}\right]}^{T},$$

(4) $$C^{\left(\mathrm{SFBC}4\right)}\left(d=\left[d_{0}\;d_{1}\;d_{2}\;d_{3}\right]\right)={\left[\begin{array}{cccc} d_{0} & d_{1} & d_{2} & d_{3}\\ -d_{1} & d_{0} & -d_{3} & d_{2}\\ -d_{2} & d_{3} & d_{0} & -d_{1}\\ -d_{3} & -d_{2} & d_{1} & d_{0}\\ d_{0}^{*} & d_{1}^{*} & d_{2}^{*} & d_{3}^{*}\\ -d_{1}^{*} & d_{0}^{*} & -d_{3}^{*} & d_{2}^{*}\\ -d_{2}^{*} & d_{3}^{*} & d_{0}^{*} & -d_{1}^{*}\\ -d_{3}^{*} & -d_{2}^{*} & d_{1}^{*} & d_{0}^{*} \end{array}\right]}^{T},$$

where *T* and * are the matrix -transpose and complex conjugate, respectively. In (1)–(4), we remove the block index *p* for ease of notation. It is important to keep in mind that *P* and *F* can have different values. In each broadcast arm, the outputs of the SFBC encoder are combined to produce a sequence $u_{\alpha }^{\left(f\right)}$ of size *N*, where $N=\frac{QN_{d}}{P}$. We have *F* broadcast arms, each of which is made up of various radio frequency circuits coupled to a transmitting antenna. We append the symbol α to the vector $u_{\alpha }^{\left(f\right)}$ to underline that the conveyed vector is dependent on the used SFBC signal $\alpha \in \left\{\mathrm{SFBC}1,\mathrm{SFBC}2,\mathrm{SFBC}3,\mathrm{SFBC}4\right\}$. Accordingly, an OFDM symbol is produced by applying an *N*-point inverse discrete Fourier transform (IDFT), and the last ν samples are included as a CP. The *n*th sample sent from the *f*th broadcast antenna is written as:

(5) $$s_{\alpha }^{\left(f\right)}\left(n\right)=\frac{1}{\sqrt{N\;+\;\nu }}\sum_{k=0}^{N-1}u_{\alpha }^{\left(f\right)}\left(k\right)e^{j2\pi nk/N},n=0,\cdots ,N\;+\;\nu -\;1$$

where $u_{\alpha }^{\left(f\right)}\left(k\right)$ is the *k*th element of sequence $u_{\alpha }^{\left(f\right)}$. The broadcast sequence

$s_{\alpha }^{\left(f\right)}={\left[s_{\alpha }^{\left(f\right)}\left(0\right),\cdots ,s_{\alpha }^{\left(f\right)}\left(N\;+\;\nu -\;1\right)\right]}^{T}$ is vulnerable to the negative effects of the IQD of the *f*th branch.

We describe $\theta^{\left(f\right)}$ and $\rho^{\left(f\right)}$ as the phase and amplitude discrepancies between I and Q segments of the *f*th arm at the broadcaster. The sequence disrupted by the IQD at arm *f* is written as [16]:

(6) $${\vec{s}}_{\alpha }^{\left(f\right)}=\eta^{\left(f\right)}s_{\alpha }^{\left(f\right)}+\mu^{\left(f\right)}s_{\alpha }^{\left(f\right)}{}^{*},$$

where $\eta^{\left(f\right)}$ and $\mu^{\left(f\right)}$ are expressed as

(7) $$\eta^{\left(f\right)}=\cos\left(\theta_{tx}^{\left(f\right)}\right)+j\rho_{tx}^{\left(f\right)}\sin\left(\theta_{tx}^{\left(f\right)}\right),$$

(8) $$\mu^{\left(f\right)}=\rho_{tx}^{\left(f\right)}\cos\left(\theta_{tx}^{\left(f\right)}\right)+j\sin\left(\theta_{tx}^{\left(f\right)}\right).$$

The sequence ${\vec{s}}_{\alpha }^{\left(f\right)}$ propagates to the destination through a wireless multipath fading channel of *L* taps, $h^{\left(f\right)}={\left[h^{\left(f\right)}\left(0\right),\cdots ,h^{\left(f\right)}\left(L-1\right)\right]}^{T}$. Therefore, the collected sequence at the destination is written as

(9) $$\bar{r}=\sum_{f=0}^{F-1}{\vec{s}}_{\alpha }^{\left(f\right)}★h^{\left(f\right)}+n,$$

where ★ is the convolution action and n is the additive white Gaussian noise (AWGN) sequence. The received signal is described here as a combination of the convolution between the signal emitted from antenna *f* and the corresponding channel coefficients, for f=0,⋯,F−1, along with the AWGN contribution. Given the influence of IQDs on the collected signal $\bar{r}$, we have

(10) $$r=\eta_{R}\bar{r}+\mu_{R}{\bar{r}}^{*},$$

where $\eta_{R}$ and $\mu_{R}$ adhere to the same pattern as those in (7) and (8). When the IQDs is taken into consideration at the receiver, the collected signal is the sum of the original received signal and its complex conjugate at different scaling factors.

Our goal is to detect the kind of SFBC signal using the intercepted signal, r, in the presence of an IQD over unidentified wireless links.

## 3. Proposed Recognition Algorithm

Using (6) and (9) in (10), one writes

(11) $$r=\sum_{f=0}^{F-1}s_{\alpha }^{\left(f\right)}★h_{1}^{\left(f\right)}+s_{\alpha }^{(f)*}★h_{2}^{\left(f\right)}+\bar{n},$$

where $\bar{n}$ is the noise contribution and

(12) $$h_{1}^{\left(f\right)}=\eta_{R}\eta^{\left(f\right)}h^{\left(f\right)}+\mu_{R}\mu^{(f)*}h^{(f)*},$$

(13) $$h_{2}^{\left(f\right)}=\eta_{R}\mu^{\left(f\right)}h^{\left(f\right)}+\mu_{R}\eta^{(f)*}h^{(f)*}.$$

Matrix representation is used to rewrite (11) for ease of calculation as

(14) $$r=\sum_{f=0}^{F-1}S^{\left(f\right)}\left(\alpha \right)h_{1}^{\left(f\right)}+S^{(f)*}\left(\alpha \right)h_{2}^{\left(f\right)}+\bar{n},$$

where $S^{\left(f\right)}\left(\alpha \right)$ is an (N+L−1)×L matrix with its item at row $x_{1}$ and column $x_{2}$ being provided as

(15) $$S_{x_{1},x_{2}}^{\left(f\right)}\;\left(\alpha \right)=\;\left\{\begin{array}{cc} s_{\alpha }^{\left(f\right)}\left(x_{1}\;-\;x_{2}\right) & \mathrm{for}x_{1}=0,\cdots ,N+L\;-\;1\\ 0 & \mathrm{for}x_{2}=0,\cdots ,L\;-\;1,x_{1}\;\geq \;x_{2}, \end{array}\right.$$

and $s_{\alpha }^{\left(f\right)}\left(x_{1}-x_{2}\right)$ is the $\left(x_{1}-x_{2}\right)$th item of sequence $s_{\alpha }^{\left(f\right)}$. The concise format of (14) is represented as

(16) $$r=\bar{S}\left(\alpha \right)\bar{h}+\bar{n},$$

where $\bar{S}\left(\alpha \right)=\left[S^{\left(0\right)}\left(\alpha \right),S^{(0)*}\left(\alpha \right),\cdots ,S^{\left(F-1\right)}\left(\alpha \right),S^{(F-1)*}\left(\alpha \right)\right]$ and

$\bar{h}={\left[h_{1}^{(0)T},h_{2}^{(0)T},\cdots ,h_{1}^{(F-1)T},h_{2}^{(F-1)T}\right]}^{T}$. The maximum likelihood prediction of α is stated as

(17) $$\hat{\alpha }=\arg\max_{\alpha }\;\log\mathrm{Pr}\left(r|\bar{S}\left(\alpha \right),\bar{h}\right),$$

with $\mathrm{Pr}\left(\circ |⋄\right)$ being the probability density function of ∘ given ⋄, and

(18) $$\mathrm{Pr}\left(r|\bar{S}\left(\alpha \right),\bar{h}\right)\propto \exp\left(-{∥r-\bar{S}\left(\alpha \right)\bar{h}∥}^{2}/\sigma_{n}^{2}\right),$$

where $∥\cdot ∥$ is the vector norm action. A straightforward development of a maximum likelihood strategy is not possible since the recipient has no previous knowledge of the broadcast matrix $\bar{S}\left(\alpha \right)$, the IQD parameters $\eta^{\left(f\right)}$, $\mu^{\left(f\right)}$, $\eta_{R}$, and $\mu_{R}$, or the channel coefficients $\bar{h}$. Expectation maximization approaches are used to determine the regional maximum likelihood parameters of a mathematical framework when the calculations cannot be solved directly. These models often include hidden variables in addition to uncertain variables and existing data inputs. Typically, obtaining a maximum likelihood strategy entails determining the derivatives of the likelihood distribution with respect to all of the unknown values, variables, and latent factors, and concurrently solving the resultant equations. Here, latent factors are the variables that can only be inferred indirectly through a mathematical model from other observable variables that can be directly observed or measured. Instead, the outcome is generally a series of interconnected formulas in which the solution to the variables necessitates the quantities of the latent factors, but inserting one series of formulas into the other yields an unsolvable problem. Convergence of an expectation maximization procedure is guaranteed because the likelihood of the predictions does not decrease. In other words, it is guaranteed to converge to a point with zero gradient with respect to the estimated parameters. An expectation maximization process consists of two stages: the expectation (E-stage) and the maximization (M-stage). Based on the predictions of the unknown variables obtained in the previous iteration *i*, the E-stage calculates the conditional mean of the log likelihood function of $\bar{S}\left(\alpha \right)$ with respect to $\bar{h}$. In mathematical terms, we write

(19) $$\begin{array}{c} U\left(\alpha ,\bar{h}\left|\hat{\alpha }\left(i\right),\hat{\bar{h}}\left(i\right)\right.\right)\;=\;E\left[\log\mathrm{Pr}\left(r,\bar{S}\left(\alpha \right)\left|r,\hat{\alpha }\left(i\right),\hat{\bar{h}}\left(i\right)\;\right.\right)\;\right]\\ \;\;\;\;\;\;=\;\int_{\bar{S}\left(\alpha \right)}\;\log\mathrm{Pr}\left(r,\bar{S}\left(\alpha \right)\;\left|r,\hat{\alpha }\left(i\right),\hat{\bar{h}}\left(i\right)\;\right.\right)\\ \;\;\;\;\;\times \mathrm{Pr}\left(\bar{S}\left(\alpha \right)\left|r,\hat{\alpha }\left(i\right),\hat{\bar{h}}\left(i\right)\right.\right)d\bar{S}\left(\alpha \right)\;. \end{array}$$

Substituting (18) into (19) while disregarding the extraneous components, the E-step is represented as

(20) $$U\left(\alpha ,\bar{h}\left|\hat{\alpha }\left(i\right),\hat{\bar{h}}\left(i\right)\right.\right)\propto -{\bar{h}}^{H}\Lambda \left(\alpha \right)\bar{h}+2ℜ\left\{r^{H}\Phi \left(\alpha \right)\bar{h}\right\},$$

where ${\left(\cdot \right)}^{H}$ is the matrix Hermitian transpose action, $ℜ\left\{\cdot \right\}$ denotes the real-part of a complex-number,

(21) $$\Lambda \left(\alpha \right)=\int_{\bar{S}\left(\alpha \right)}{\bar{S}}^{H}\left(\alpha \right)\bar{S}\left(\alpha \right)\mathrm{Pr}\left(\bar{S}\left(\alpha \right)\left|r,\hat{\alpha }\left(i\right),\hat{\bar{h}}\left(i\right)\right.\right)d\bar{S}\left(\alpha \right),$$

and

(22) $$\Phi \left(\alpha \right)=\int_{\bar{S}\left(\alpha \right)}\bar{S}\left(\alpha \right)\mathrm{Pr}\left(\bar{S}\left(\alpha \right)\left|r,\hat{\alpha }\left(i\right),\hat{\bar{h}}\left(i\right)\right.\right)d\bar{S}\left(\alpha \right).$$

M-stage revises the predictions as

(23) $$\left[\hat{\alpha }\left(i+1\right),\hat{\bar{h}}\left(i+1\right)\right]=\arg\max_{\alpha ,\bar{h}}\;U\left(\alpha ,\bar{h}\left|\hat{\alpha }\left(i\right),\hat{\bar{h}}\left(i\right)\right.\right).$$

Here, we obtain the revised estimates by maximizing the expectation function with respect to α and $\bar{h}$, using the known baseline values for those parameters. We design the following tactic to ease the execution of the two-dimensional optimization process shown in (23). We revise $\bar{h}$ by optimizing (20) for each possible element of α as

(24) $${\hat{\bar{h}}}_{\alpha }\left(i+1\right)={\left(\Lambda \left(\alpha \right)\right)}^{-1}{\left(\Phi \left(\alpha \right)\right)}^{H}r.$$

Inserting (20) and (24) into (23), the revised value of α is represented as

(25) $$\begin{array}{ccc} \hat{\alpha }\left(i+1\right) & \;=\; & \arg\max_{\alpha }\left\{-{\hat{\bar{h}}}_{\alpha }^{H}\left(i+1\right)\Lambda \left(\alpha \right){\hat{\bar{h}}}_{\alpha }\left(i+1\right)\right.\\ & \;\;\;\;\;\; & \;\;\;+\left.2ℜ\left\{r^{H}\Phi \left(\alpha \right){\hat{\bar{h}}}_{\alpha }\left(i+1\right)\right\}\right\}. \end{array}$$

The last channel adjustment is acquired as

(26) $$\hat{\bar{h}}\left(i+1\right)={\hat{\bar{h}}}_{\hat{\alpha }}\left(i+1\right).$$

**Figure 2:**
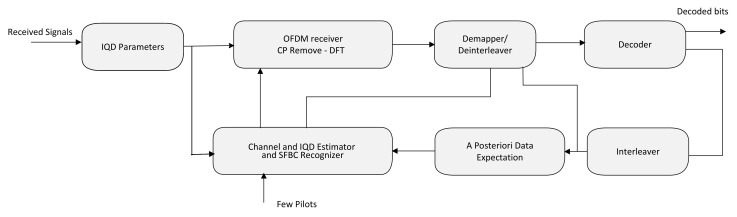
The basic structure of the solution being offered. The main contributing blocks are the channel and IQD estimator and SFBC recognizer along with a posteriori data expectation.

Figure 2 shows the basic structure of the offered solution. It should be noted that the proposed approach necessitates the participation of a few pilots in order to provide preliminary estimates of the unknown parameters. The following concerns pertaining to reality are of significance:

1. We have demonstrated, depending on (5), that
  (27) $$\begin{array}{c} E\left[s_{\alpha }^{\left(f\right)}\left(n\right)\left|r,\hat{\alpha }\left(i\right),\hat{\bar{h}}\left(i\right)\right.\right]=\frac{1}{\sqrt{N\;+\;\nu }}\sum_{k=0}^{N-1}\\ \;\;E\left[u_{\alpha }^{\left(f\right)}\left(k\right)\left|r,\hat{\alpha }\left(i\right),\hat{\bar{h}}\left(i\right)\right.\right]e^{j2\pi nk/N}, \end{array}$$
  where
  (28) $$\begin{array}{c} E\left[u_{\alpha }^{\left(f\right)}\left(k\right)\left|r,\hat{\alpha }\left(i\right),\hat{\bar{h}}\left(i\right)\right.\right]=\\ \;\;\sum_{\varpi \in \Omega }\varpi \mathrm{Pr}\left[u_{\alpha }^{\left(f\right)}\left(k\right)=\varpi \left|r,\hat{\alpha }\left(i\right),\hat{\bar{h}}\left(i\right)\right.\right]. \end{array}$$
  Here, ϖ denotes each possible point in the modulation constellation Ω. In reality, rather than relying on the mysterious $s_{\alpha }^{\left(f\right)}\left(n\right)$ supplied by the transmitter, the recipient utilizes $E\left[s_{\alpha }^{\left(f\right)}\left(n\right)\left|r,\hat{\alpha }\left(i\right),\hat{\bar{h}}\left(i\right)\right.\right]$ to generate the matrix of $\Phi \left(\alpha \right)$. Moreover, because an interleaver is present, Λ(α) is efficiently calculated as $\Phi^{H}\left(\alpha \right)\Phi \left(\alpha \right)$.
2. It has been noted that determining $\mathrm{Pr}\left[u_{\alpha }^{\left(f\right)}\left(k\right)=\varpi \left|r,\hat{\alpha }\left(i\right),\hat{\bar{h}}\left(i\right)\right.\right]$ is essential for the suggested recognizer. This likelihood is determined from the decoder findings of any error control coding scheme that can establish soft representations. More information about this concern is provided in [39].
3. Sending pilot symbols from the broadcaster to the destination provides the proposed estimator and recognizer with a starting point. With more known symbols, the original assessment becomes more accurate. Nevertheless, more known symbols means less power is accessible for information and implies more bandwidth is needed. Consequently, it is essential that there be as few known symbols as possible in comparison to data symbols. The offered expectation maximization method is effective for this purpose because it treats the results provided by the channel decoders as if they were already known symbols. This incrementally enhances the first estimate without adding more training symbols. Using a small number of training symbols while initializing the unknown data symbols at zero, we obtain first-order predictions of the uncertain variables. The suggested classifier is derived via a series of rounds that use the soft outputs given by the error control decoder to produce $\mathrm{Pr}\left[u_{\alpha }^{\left(f\right)}\left(k\right)=\varpi \left|r,\hat{\alpha }\left(i\right),\hat{\bar{h}}\left(i\right)\right.\right]$. It is well known that all contemporary decoding procedures produce a posteriori probabilities of the transmitted symbols for use in their iterative frameworks. The proposed recognition and estimation approach exploits these probabilities to produce expected values of the transmitted symbols, which are employed as if they were known symbols.
4. By adopting a computational complexity analysis as stated in [26,33], we demonstrate that the count of floating point operations (flps) requested for a single iteration is represented by
  (29) $$\rho =\sum_{F\in \{2,2,3,4\}}\;\;\;24L^{2}F^{2}\left(N+\nu +L-1\right)+4L^{3}F^{3}.$$
  Consider N=512, L=7, ν=6, and a processor that runs at 10 Teraflps per second, then $\rho =20.5\times {\left(10\right)}^{6}$ flps. This necessitates a computation time of 2.05 μs, which seems to be insignificant for real situations.

## 4. Simulation Results

The recognition efficiency of the offered approach was evaluated via Monte Carlo simulations. Unless otherwise declared, we assume that the OFDM scheme is operating at N=512, ν=7, and 16 QAM. The SFBC collection under examination is {SFBC1, SFBC2, SFBC3, and SFBC4} with their broadcast matrices being defined as indicated in (1)–(4).

A convolutional code is adopted with a rate of 1/2, 16 states memory elements, and generator polynomials of 32 and 37 with a base of 8 [40]. The suggested recognizer is launched utilizing 70 dispersed known symbols. The proposed method is flexible because there are no predetermined positions for the pilot symbols. The radio connection between each broadcast and receiver antenna is assumed to be multiple taps, with a length of L=6 and a power delay profile of

(30) $$\sigma^{\left(f\right)2}\left(l\right)=\varrho \exp\left(-l/6\right),$$

where ϱ is chosen to have a mean power per subcarrier of one [39]. The IQD parameters of the broadcaster and receiver are chosen at random as follows: $\rho_{tx}^{\left(f\right)},\rho_{rx}\in \left[0.92,1.5\right]$, and $\theta_{tx}^{\left(k\right)},\theta_{rx}\in \left[0,20^{o}\right]$. The probability of false recognition $P_{f}$ is utilized as an efficiency indicator for the suggested recognizer,

(31) $$\begin{array}{c} P_{f}=1-\left[\mathrm{Pr}\left(\mathrm{SFBC}1\left|\mathrm{SFBC}1\right.\right)+\mathrm{Pr}\left(\mathrm{SFBC}2\left|\mathrm{SFBC}2\right.\right)\right.\\ \;\;+\left.\mathrm{Pr}\left(\mathrm{SFBC}3\left|\mathrm{SFBC}3\right.\right)+\mathrm{Pr}\left(\mathrm{SFBC}4\left|\mathrm{SFBC}4\right.\right)\right]. \end{array}$$

Note that the previous formulation of $P_{f}$ was constructed assuming that four SFBC signals are used. For any other set of SFBC signals, an equivalent expression can be simply obtained. In addition, the bit error rate (BER) is used as a quality metric of the entire system, whereas the mean square estimation error (MSE) is utilized to assess the offered estimation of the IQD parameters and radio links.

**Figure 3:**
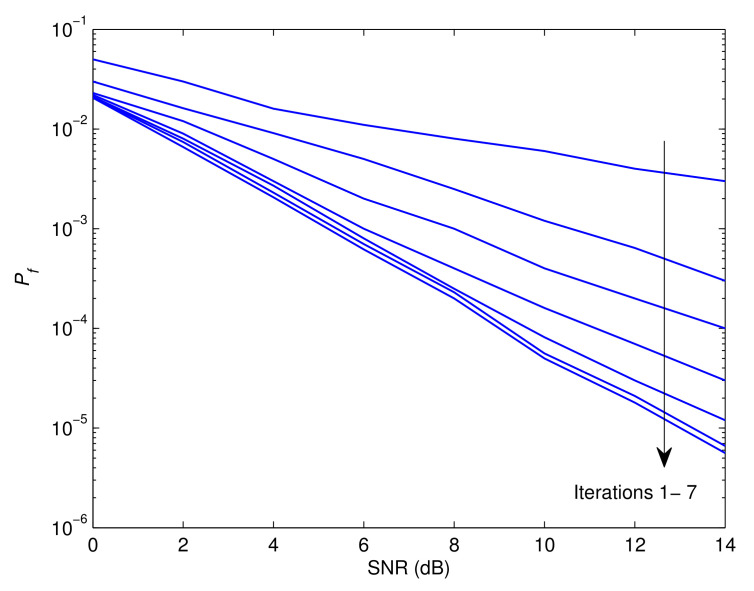
Probability of false recognition of the proposed recognizer as a function of the SNR.

**Figure 4:**
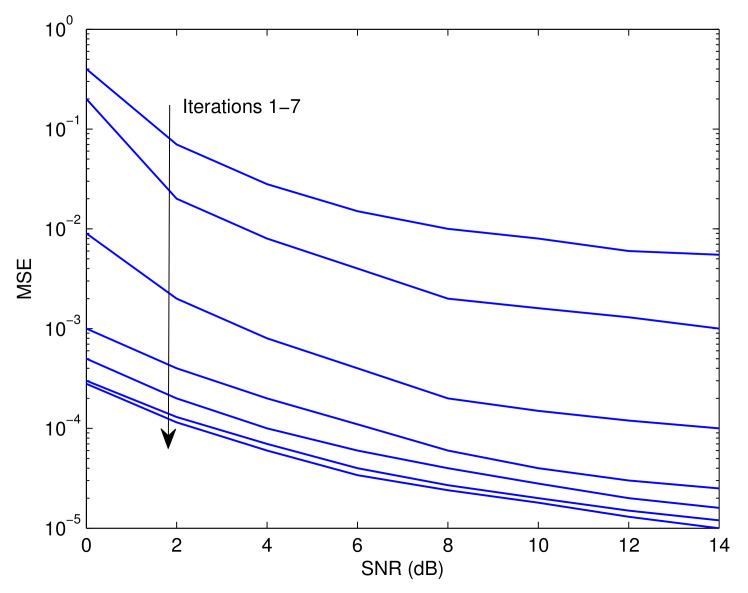
Mean square estimation error of the proposed estimator as a function of the SNR.

**Figure 5:**
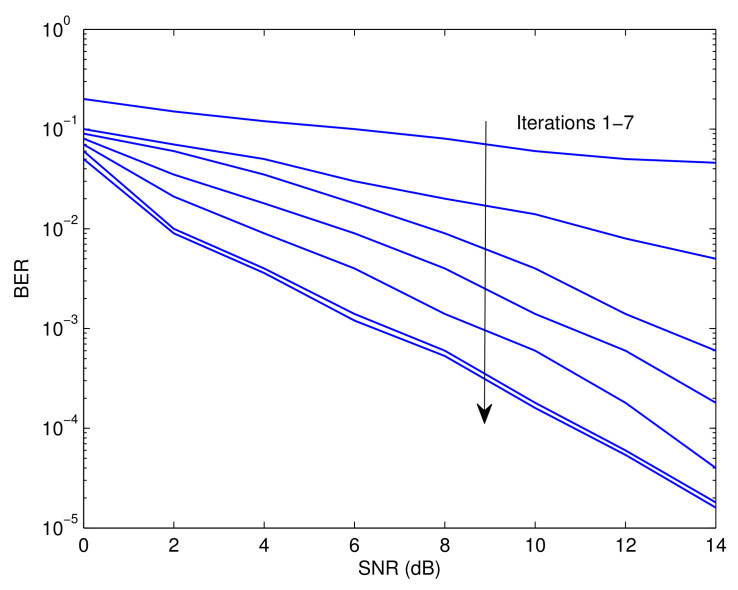
Bit error rate of the proposed approach as a function of the SNR.

Figure 3, Figure 4 and Figure 5 illustrate the $P_{f}$, MSE, and BER, respectively, of the proposed approach as a function of the signal-to-noise ratio (SNR). Observably, the effectiveness of the offered strategy improves as the iteration process proceeds. The results reveal that the proposed method converges after around seven iterations. The following is an explanation for this tendency. The detector’s soft information is imprecise in the first round since the recognition and prediction methods are based on a small number of known symbols. In contrast to the data-assisted situation, the estimate and recognition accuracies rise with an increase in the number of iterations since more reliable information is incorporated by the error control decoder. Furthermore, it is evident that the first five iterations generate the best and most efficient results with minimal delay. In practice, the mode of operation with the smallest delay and the highest impact could be attributed to the fourth iteration. This is also consistent with the results shown in [41].

**Figure 6:**
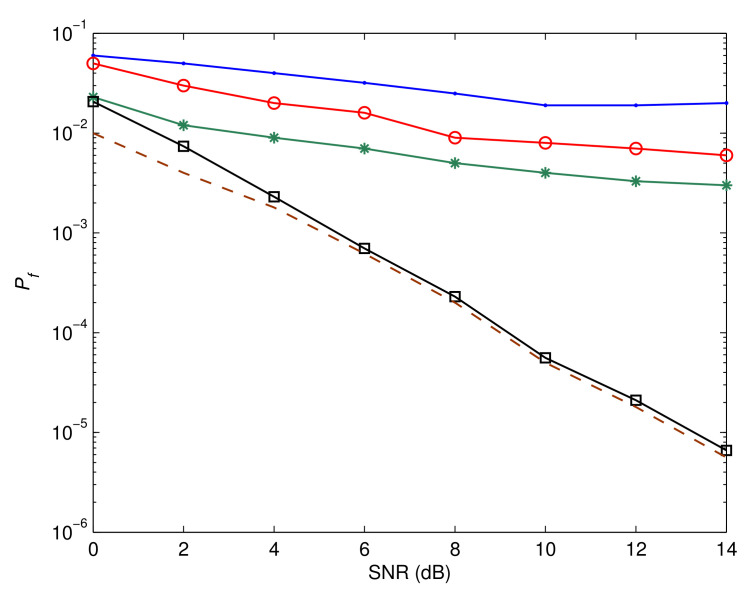
Probability of false recognition comparison. The blue line represents the performance without correcting IQD, red line shows the performance of the algorithm reported in [42], green line describes the performance of the algorithm reported in [43], black line shows the performance of the proposed algorithm, and brown line indicates the benchmark performance.

**Figure 7:**
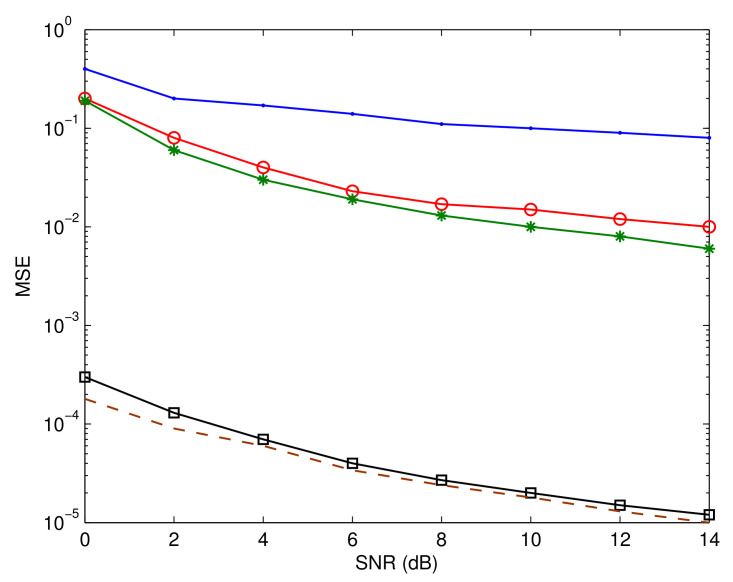
Mean square estimation error comparison. The blue line represents the performance without correcting IQD, red line shows the performance of the algorithm reported in [42], green line describes the performance of the algorithm reported in [43], black line shows the performance of the proposed algorithm, and brown line indicates the benchmark performance.

**Figure 8:**
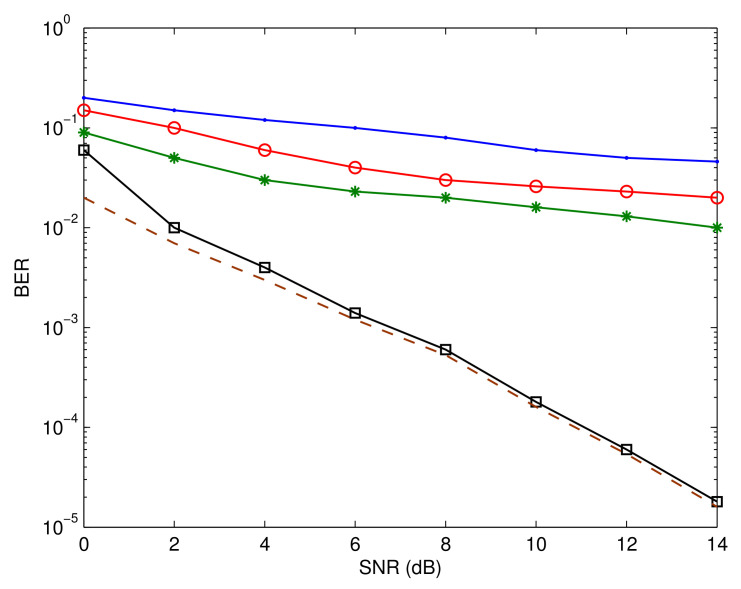
Bit error rate comparison. The blue line represents the performance without correcting IQD, red line shows the performance of the algorithm reported in [42], green line describes the performance of the algorithm reported in [43], black line shows the performance of the proposed algorithm, and brown line indicates the benchmark performance.

Figure 6, Figure 7 and Figure 8 depict $P_{f}$, MSE, and BER, respectively, of a number of different systems for the sake of comparison. The first system is based on the assumption that the IQD parameters are not be calculated and adjusted. The performances of the systems introduced in [42,43] are also shown. Furthermore, we highlight the system’s efficiency under the presumption that its input data are completely known. This functions as a benchmark for the proposed estimation and recognition processes. In addition, we display the BER performance under perfect circumstances where unknown parameters can be precisely estimated and identified. As is evident, the performance is poor if the IQD is not resolved. The suggested method also has a substantial performance advantage over the methods presented in [42,43]. This is due to the fact that the suggested method compensates for IQDs, while the aforementioned methods do not. Moreover, the suggested estimation and recognition iterative approach accomplishes a BER performance that is comparable to that of the ideal system.

**Figure 9:**
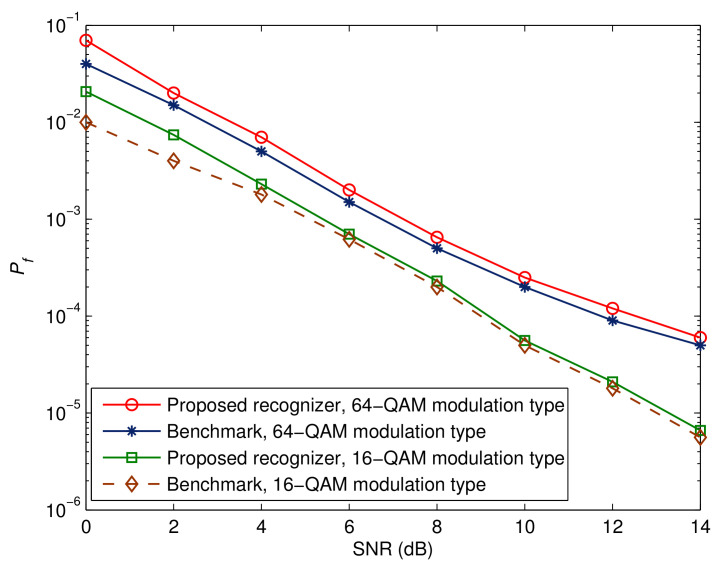
Probability of false recognition for different modulation types.

**Figure 10:**
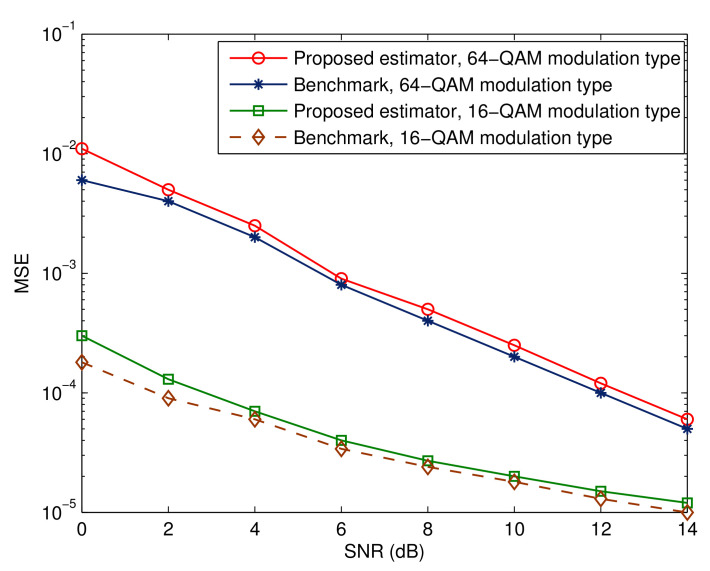
Mean square estimation error for different modulation types.

**Figure 11:**
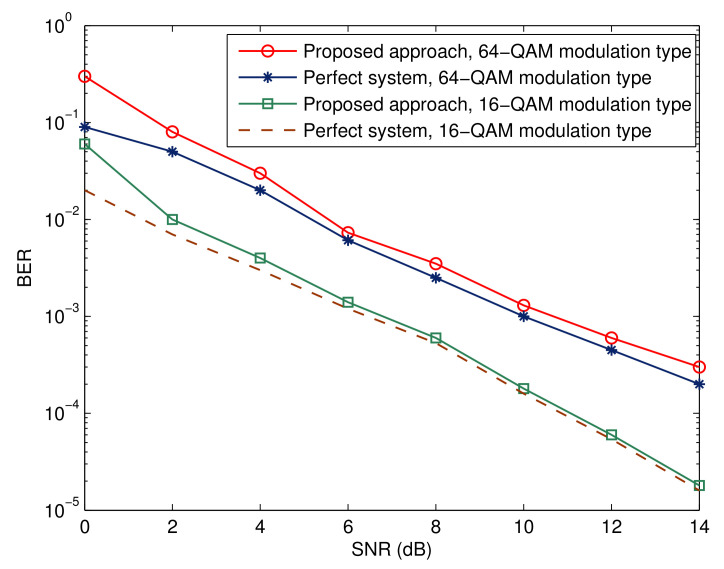
Bit error rate for different modulation types.

Figure 9, Figure 10 and Figure 11 depict the effectiveness of the advocated technique with respect to various modulation kinds. We also show the corresponding benchmarks and BER performance in the ideal situations. Two points are worth mentioning. The first point is that there is a decline in performance when higher-order modulation types are adopted. This is due to the fact that higher-order modulation formats result in less accurate soft information representations from the error control decoder, hence reducing the maximum possible performance. The second observation is that the performance of the suggested method is within 1 dB of the relevant benchmarks. This proves that the suggested receiver architecture is beneficial.

## 5. Conclusions and Future Work

The identification of SFBC waveforms was considered for OFDM broadcasts in the setting of IQD parameters over anonymous radio links. We integrated the radio links into the IQD effects at both ends of the transmission chain to form equivalent wireless connections. The offered method simultaneously recognizes SFBC waveforms, calculates the IQM at each station, and monitors channel taps across broadcaster and receiver antennas, eliminating the requirement to employ several algorithms. We developed an original maximum likelihood technique for assessing the relevant parameters. In order to efficiently implement the stated technique, we used an expectation maximization iterative approach. The following are various benefits that result from our suggested method.

- The stated method makes use of the iterative nature of contemporary error control decoders. To be more precise, the soft information produced by the error control detector is leveraged in an iterative manner to enhance the estimation and recognition processes. Moreover, it can function with any detection process, as the detector is capable of computing the a posteriori probabilities of the information bits.
- The proposed algorithm can be utilized in place of many independently operating algorithms to predict the sender and receiver IQD parameters, to estimate the channel taps, and to recognize SFBC signals.
- It can be employed with any collection of SFBC signals, regardless of the form their transmission matrices may take.
- It keeps the degree of computational expenditure at a bearable level while maintaining an outstanding performance.

In simulations, using the suggested recognizer in tandem with the supplied estimator yielded results extremely near to those obtained in the perfect scenario when all of the parameters were available in advance. The suggested approach also performed better than the state-of-the-art algorithms described in the literature. The following is a list of prospective future work.

- The current recognition and estimation algorithm needs a quasi-static link over the emitted frame period. Modifying the algorithm to accommodate time-varying channels requires additional research.
- Additional exploration is required to develop SFBC recognition algorithms in the presence of other transmission impairments, such as frequency offsets and phase noise.

## Data Availability

Not applicable.
